# Associated symptoms of depression: patterns of change during pregnancy

**DOI:** 10.1007/s00737-016-0685-6

**Published:** 2016-11-22

**Authors:** Rita T. Amiel Castro, Claudia Pinard Anderman, Vivette Glover, Thomas G. O’Connor, Ulrike Ehlert, Martin Kammerer

**Affiliations:** 1 Imperial College London, Faculty of Medicine, Institute of Reproductive and Developmental Biology, Du Cane Road, London, W12 ONN UK; 2 Department of Clinical Psychology and Psychotherapy, Institute of Psychology, University of Zurich, Zurich, Switzerland; 3 Department of Applied Psychology, University of Applied Sciences Zurich, Winterthur, Switzerland; 4 Department of Psychiatry, University of Rochester Medical Centre, Rochester, NY USA

**Keywords:** Pregnancy, Somatic symptoms, Psychological symptoms, Monthly patterns

## Abstract

Little is known about the natural course of depressive symptoms and associated features throughout pregnancy. We examined the course of some psychological and somatic symptoms in each month of pregnancy in a normative sample. A consecutive, unselected sample of women (*N* = 374) were interviewed retrospectively at 6 weeks postpartum with the Structured Clinical Interview (DSM-IV). Women were asked whether they had experienced each symptom at any time during pregnancy and the occurrence of the symptom for each month of pregnancy. Associated symptoms of depression showed complex changes across pregnancy. Depressed mood (*F*(df) = 5.15(1); *p* = 0.02) showed a quadratic pattern with elevations at the beginning and end of pregnancy. Both linear increases (^a^) and quadratic (^b^) changes over time were observed for sensitivity to criticism (*F* ^a^(df) = 20.9(1), *p* ^a^ = 0.00; *F* ^b^(df) = 7.02(1), *p* ^b^ = 0.00), lack of concentration (*F* ^a^(df) = 37.0(1), *p* ^a^ = 0.00; *F* ^b^(df) = 10.3(1); *p* ^b^ = 0.00), decreased energy (*F* ^a^(df) = 13.4(1); *p* ^a^ = 0.00; *F* ^b^(df) = 62.6(1); *p* ^b^ = 0.00) and feelings of heavy limbs (*F* ^a^ (df) = 92.9(1); *p* ^a^ = 0.00; *F* ^b^(df) = 67.7(1); *p* ^b^ = 0.00). Only guilt (*F*(df) = 0.00(1); *p* = 0.93) showed no significant change over pregnancy. Psychological symptoms changed throughout pregnancy as much as somatic symptoms. A linear increase was found for most symptoms, but significant non-linear changes were also found. The discrepancy between the patterns of depressed mood and most somatic and psychological symptoms suggest complex interactions and potentially important implications for assessment and monitoring treatment.

## Introduction

Pregnancy, birth and lactation are marked by large changes in hormone levels, including increasing exposure to psychoactive hormones such as oestrogen, progesterone and cortisol during pregnancy (O’Leary et al., 1991; Yonkers et al., 2009) and sudden withdrawal on parturition (Kammerer et al., 2009). There is considerable interest in examining these and other periods of marked hormonal change (e.g. pubertal, menopause) to gain insight into the possible hormonal basis of psychiatric symptoms, both prior to and past the perinatal period (Winkel et al. 2013).

Dramatic changes in hormone levels in pregnancy might be expected to predict changing depressive symptoms. For example, at their peak, oestrogen levels are 30 times higher in pregnancy than during the menstrual cycle and cortisol levels in pregnancy are as high as in major depressive disorders (Glynn, 2012). The hormonal rise throughout pregnancy may promote the emergence of somatic and depressive symptoms (Lommatzsch et al., 2006; Banti et al., 2011), but there are surprisingly few studies that systematically assess changes in depressive symptoms over the course of pregnancy. Research has shown that amplification of certain somatic symptoms among pregnant women may be associated with prenatal depressive disorders (Kelly et al. 2001; Anderson et al. 2003). Thus, the current study adds to existing research by charting the patterns of depressive symptoms and associated features over the entire course of pregnancy in a large, non-selected group of pregnant women.

There is evidence that depression increases with or at least varies across gestation (Evans et al. 2001; Bennett et al. 2004). However, there are two potentially important limitations of the existing literature. One is failure to distinguish among different kinds of depressive symptoms, most typically because the measure is based on a composite self-report questionnaires or a diagnosis of depression. It may be that this broad-based measure masks considerable changes in the component symptoms of depression, including somatic symptoms associated with energy and fatigue, and specific psychological symptoms associated with self-concept and self-regard. Assessing specific symptoms of depression in detail may be a more sensitive approach to determining if different symptoms show differential patterns across gestation. Results of this more intensive longitudinal assessment may yield practical lessons about management of symptoms at different points in pregnancy and refine the search for links between hormonal changes and behavioural symptoms (although the specificity of the links between a specific biological change and clinical presentation is not well established). The second limitation is that most studies are limited to two or three assessments, usually from mid- to late pregnancy. That is significant because tracing the connections between depressive associated symptoms and hormonal changes will be best informed by a consideration of the wide variation from the beginning to the end of pregnancy.

We examined the course of both psychological (depressed mood, poor self-esteem, guilt, lack of concentration, sensitivity to criticism, thoughts of death) and some somatic (decreased energy, feelings of heavy limbs and feeling worse in the morning) symptoms in each month of pregnancy in a non-clinical population from a clinical interview. We hypothesise that psychological and somatic symptoms will vary across pregnancy and that this may be due to naturally occurring changes in hormones and/or psychological changes associated with the impending birth; given the very limited available literature, the nature of the changes in clinical presentation was largely exploratory. Novel and important methodological features of the study include assessing multiple expressions of both psychological and somatic symptoms and tracking symptoms through all 9 months of gestation.

## Material and methods

### Participants

Of 672 women who were approached, 374 consecutive women were interviewed and provided complete information about somatic and psychological symptoms experienced in pregnancy. All eligible participants who decided to take part in this study provided complete data. Eligible participants who decided not to take part in the study (*n* = 298) provided only basic data such as age, parity, number of pregnancies, marital status and job, anonymously. Participants and eligible non-participants did not show significant sociodemographic differences. Participants were recruited between 2003 and 2006 from five obstetric hospital units in the Canton of Zurich, Switzerland, which offered their services to the city, suburban and rural catchment areas (e.g. Knonauer Amt, Zurich Unterland) being responsible for half of the annual birth rate of the canton. Consecutive women were recruited at 4 days postpartum to participate in this study. Exclusion criteria were psychotic features, current drug or alcohol addiction and poor general medical condition. Participants on psychotropic medication or any psychological treatment during the perinatal were excluded from analysis because of our interest in normative changes. Participants were asked for written informed consent to be part of this study. Ethics approval from the Canton of Zurich was obtained before the commencement of the study.

### Procedure

Data regarding monthly pregnancy symptoms were obtained retrospectively from all women who endorsed any symptoms during any time in pregnancy. At 6 weeks postpartum, trained researchers (psychology, psychiatry and midwifery area) carried out the Structured Clinical Interview for DSM IV (Diagnostic and Statistical Manual for Mental Disorders IV, German version (First et al. 2002)). The SCID interview was carried out via telephone and all symptoms reported here are part of the DSM-IV as associated symptoms of depression. Sociodemographic data as well as medical information and history of drug abuse were also collected from participants.

Interrater reliability was assessed with 50 tape recorded and repeated interviews randomly selected. The interviewer’s judgement and the training psychiatrist’s judgement were correlated 0.68–0.82, kappa coefficient (Cohen 1960).

### Measures

The SCID interview is specifically designed to assess diagnoses of DSM IV. If the interviewee does not qualify for the entry criteria (severity and duration of depressive mood and loss of interest) of a depressive episode, the interview is finished. In this study, a modified version of the SCID interview for DSM IV was employed that allowed assessment of some DSM IV symptoms without regard to presence of depressed mood and anhedonia. That is, all participants were assessed for SCID symptoms irrespective of whether they fulfilled the SCID entry criteria. One further adaptation concerning timing was required to assess symptoms throughout pregnancy. Women were asked if they experienced each symptom at any time in pregnancy (i.e. rather than the past 2 weeks); women who endorsed experiencing the symptom at any point in pregnancy were then asked about the occurrence for each month in pregnancy. The questions asked were as follows: “Was there a time during your pregnancy where you felt depressed or sad almost every day?”, “During your pregnancy, have you felt you lost your energy, felt constantly tired or exhausted?, “Have you had difficulty thinking or concentrating?”, “About your self-esteem, did you feel worthless?”, “Many people have during a difficult time, thoughts of death, was this the case with you?”, “During your pregnancy, did you usually feel worse in the morning?”, “Did you feel guilty about things you have done or not done? “Did you feel your arms or legs were leaden sometimes?”, “Did you react particularly sensitively to how others treated you?”

Participants’ occupation was divided in three categories: professional/managerial position; skilled (manual and non-manual) position and unskilled position. None of the participants had an unskilled position and none of them declared to be unemployed. However, women who reported to be a housewife were considered unemployed.

### Statistical analysis

Repeated measures of general linear model were used to analyse each symptom in all 9 months of pregnancy. Tests of within-subject contrast were analysed as well as inter-item correlation. Cronbach’s alpha, to determine internal consistency, was obtained. A bivariate analysis was carried out to measure the association between the symptom of depressed mood and all the other symptoms throughout the 9 months of pregnancy. Associations between maternal age, SES, parity and smoking and changes in all symptoms over time were analysed with repeated measures analysis of covariance (ANCOVA) including time versus symptoms interaction.

## Results

Characteristics of the participants are shown in Table 1. Repeated measures ANCOVA showed no significant association between any of the psychological and somatic symptoms with age, SES, parity and smoking.

**Table 1:** Sociodemographic characteristics of the sample (*n* = 374)

	Mean	SD/range	Percent
Age	31.9	4.9/27	–
Parity	1.5	0.8/7.0	–
Number of pregnancies	1.84	1.18/9.0	–
Baby sex (M)	–	–	52.2
Marital status (married)	–	–	72.8
Smoking in pregnancy (any)	–	–	18.5
Drug use in pregnancy (any)	–	–	2.4
Alcohol in pregnancy (any)	–	–	1.1
Occupation	–	–	
Professional/managerial position			48.8
Skilled (manual and non-manual) position			32.2
Unskilled position			–
Unemployed			19.0
Ethnicity (Caucasian)	–	–	>97

*SD* standard deviation, *M* male

**Fig. 1:**
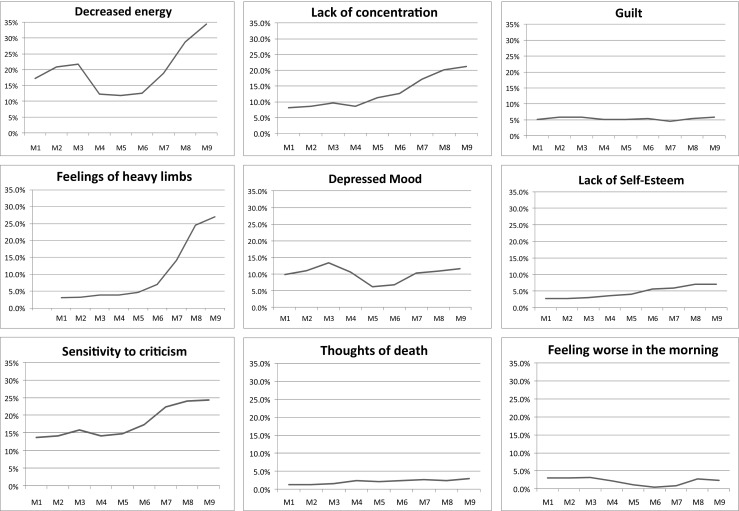
Patterns of change of psychological and somatic symptoms in pregnant women in each month of pregnancy (*n* = 374)

Figure 1 shows the month-by-month pattern for the symptoms studied. Most symptoms showed significant linear increases over time. In addition, most symptoms (depressed mood, feeling worse in the morning, decreased energy, lack of concentration, sensitivity to criticism, feelings of heavy limbs) also showed a quadratic pattern with symptoms higher at the beginning and end of pregnancy than in the middle. Sensitivity to criticism (*F* ^a^(df) = 20.9(1); *p* ^a^ = 0.00; *F* ^b^(df) = 7.02(1); *p* ^b^ = 0.00), lack of concentration (*F* ^a^(df) = 37.0(1); *p* ^a^ = 0.00; *F* ^b^(df) = 10.3(1); *p* ^b^ = 0.00), decreased energy (*F* ^a^ (df) = 13.4(1); *p* ^a^ = 0.00; *F* ^b^(df) = 62.6(1); *p* ^b^ = 0.00) and feelings of heavy limbs (*F* ^a^(df) = 92.9(1); *p* ^a^ = 0.00; *F* ^b^(df) = 67.7; *p* ^b^ = 0.00) showed both linear (^a^) and quadratic (^b^) patterns. Feelings of guilt (*F*(df) = 0.00(1); *p* = 0.93) did not change significantly across time; feeling worse in the morning (*F*(df) = 8.39(1);*p* = 0.00) and depressed mood (F(df) = 5.15(1); *p* = 0.02) showed only a quadratic pattern, and thoughts of death (*F*(df) = 4.52(1); *p* = 0.03) and poor self-esteem (*F*(df) = 10.15(1); *p* = 0.00) showed only a linear pattern over the course of pregnancy.

Women varied in which symptoms they experienced and how much they experienced a particular symptom. Only about 5 % experienced feelings of guilt and only about half of that had thoughts of death. But by the end of pregnancy, 30–35 % of women had feelings of heavy limbs and decreased energy, about 25 % were particularly sensitive to criticism, and over 20 % had problems with concentration.

To measure internal consistency reliability, inter-item correlations and Cronbach’s alpha were obtained. Inter-item correlations showed weak values across all 9 months of pregnancy as well as Cronbach’s alpha that presented the lowest value α 0.339 in month 1 and the highest α 0.479 in month 7.

The association between feelings of depression and all the other symptoms studied in each month is shown in Table 2. In general, the associations ranged from Φ = −0.03 to 0.22. The strongest associations were between feelings of depression and decreased energy (Φ = 0.22 in month 6) and depression and sensitivity to criticism (Φ = 0.22 in month 3).

**Table 2:** Relationship between depressed mood and each associated symptom per pregnancy month (bivariate analysis; *p < 0.05 significant, *n* = 374)

	Depressed	Mood								
Symptoms		1	2	3	4	5	6	7	8	9
		Phi p	Phi p	Phi p	Phi p	Phi p	Phi p	Phi p	Phi p	Phi p
1. Lack of concentration		0.032 0.53	0.009 0.862	0.031 0.55	0.083 0.110	0.014 0.78	0.027 0.60	0.11 0.83	0.85 0.101	0.100 0.05*
2. Decreased energy		0.86 0.095	0.062 0.235	0.117 0.02*	0.169 0.00*	0.186 0.00*	0.225 0.00*	0.090 0.08	0.108 0.03*	0.112 0.03*
3. Guilt		0.077 0.137	0.055 0.289	0.065 0.20	−0.40 0.446	0.009 0.864	0.31 0.54	0.011 0.82	0.044 0.393	0.055 0.289
4. Feelings of heavy limbs		0.104 0.046	0.079 0.130	*0.173 0.00* *	0.116 0.025	0.050 0.33	0.095 0.06	0.145 00*	0.084 0.106	0.121 0.02*
5. Feeling worse in the morning		0.101 0.051	0.185 0.00*	0.151 0.00*	0.131 0.012	0.081 0.116	0.020 0.70	0.069 0.18	0.103 0.04*	0.107 0.03*
6. Lack of self-esteem		0.111 0.032	0.148 0.00*	0.117 0.02*	0.174 0.001	0.118 0.02*	0.120 0.02*	0.066 0.20	0.109 0.03*	0.165 0.00*
7. Sensitivity to criticism		0.211 0.00*	0.218 0.00*	0.222 0.00*	0.090 0.083	0.011 0.83	0.020 0.70	0.142 0.00*	0.151 0.00*	0.130 0.01*
8. Thoughts of death		–0.039 0.45	0.042 0.416	0.012 0.81	0.117 0.024	0.116 0.02*	0.028 0.594	0.109 0.03*	0.058 0.261	0.036. 486

The italicized results are statistically significant

## Discussion and conclusion

This study has shown that both psychological and somatic symptoms change during pregnancy, but in different patterns (Fig. 1). In general, there was a low correlation between depressed mood and other symptoms (Table 2). Women also varied considerably in the degree to which they showed each specific symptom; for instance, by the end of pregnancy, 35 % reported feelings of heavy limbs and decreased energy, and 25 % reported being unusually sensitive to criticism (Fig. 1); 12 % had endorsed depressed mood. The current study is different in design from previous studies concerning changes in depression during pregnancy. We asked all women about a range of their symptoms throughout pregnancy, not only those with a diagnosis of depression; the findings therefore extend more broadly and reflect the general course of symptoms. Our finding of 12 % of participants experiencing depressed mood, together with the evidence of a quadratic pattern throughout pregnancy, is consistent with prior reports of higher levels of psychological symptoms in the first and third gestational trimester (Martini et al. 2013).

The marked changes in most symptoms that can be associated with depression from the first to the ninth month of pregnancy is a novel finding. This may reflect the changing biology in pregnancy and well as psychological changes and adjustments to the impending delivery. We did not have sufficient data to link specific symptoms to individual biological or psychosocial factors, but previous research suggests several targets for further study. A natural target for further research is oestrogen and progesterone, which have neuroregulatory effects, including on the central serotonin system (Moses-Kolko et al. 2008), and have been implicated in psychological symptoms in the perinatal period (Maccaria et al. 2003; Bloch et al. 2003). Also, Fan et al. (2009) found that rates of anxiety and depression were higher in the first trimester than later in pregnancy and that depression was correlated with changes in estradiol and progesterone level, and anxiety was correlated with total cortisol level. Winkel et al. (2013) found, in a general population, that premenstrual symptoms were associated with both psychological and physical symptoms in the first pregnancy trimester. They concluded that this finding supports the hormonal sensitivity hypothesis, that some women are prone to experience specific psychological and physical symptoms during different reproductive phases. These findings are of considerable interest. However, we note that the complexity of the pattern of symptoms found here over the whole course of pregnancy—that is, quadratic pattern for some symptoms but linear patterns for others—does not easily match what is known about pregnancy-related changes in and interactions among psychoactive hormones in pregnancy.

Previous authors have discussed whether somatic symptoms during pregnancy may unnecessarily bias the diagnosis of depression. For example, Klein and Essex (1994) studied symptoms in the second trimester and concluded that the overlap between symptoms of depression and complaints associated with pregnancy may bias estimates of depression in studies of women of childbearing age. On the other hand, Nylen et al. (2013) found that fatigue and sleep disruptions are effective indicators of depression in pregnant women and are an important part of the depressive symptomatology in pregnant women and could be as incapacitating as psychological symptoms (Nylen et al. 2013; Manber et al. 2008). Our analyses of the changing covariation between, i.e. depressed mood and somatic symptoms across the 9 months of pregnancy indicate it may be clinically more useful to consider how the meaning of symptoms change over gestation rather than simply dismiss certain symptoms as uninformative regarding depression for any pregnant woman.

The current study has several limitations. First, the collection of information about the symptoms in pregnancy was retrospective. This is the norm with the SCID interview, but the current study assessed a longer time period and specificity across time periods. Participant recall bias is an important possible source of error, although is unlikely to explain the recall of different pattern of change for the different symptoms and the non-linear nature of the pattern for many symptoms. Also, it is important to note that a prospective design requiring monthly assessments is rather burdensome and might introduce bias in reporting too, e.g. by making women more self-conscious of symptoms over time. Another limitation was the exclusion of women using psychotropic medication or receiving psychotherapeutic treatment. This might have excluded women with very elevated symptoms, but does mean that we were able to track the normative course of symptoms throughout pregnancy. In addition, we did not use data on other symptoms that are associated with depression, such as eating and sleep patterns. These have been considered to be potentially unreliable as an associated symptom of atypical depression during pregnancy (for a review see: Kammerer, et al., 2011). However, in a future study of this nature, it would be of interest to examine the pattern of change of these symptoms also. Despite these limitations, the current study does suggest that it is of interest to look in detail at the pattern of change in a range of symptoms that can be associated with depression, over the whole course of pregnancy. Despite these limitations, the current study does suggest that it is of interest to look in detail at the pattern of change in a range of symptoms that can be associated with depression, over the whole course of pregnancy.

These results have potential implications for the assessment and treatment of depression during pregnancy. These normative changes in symptom expression may aid in tracking and interpreting symptoms for clinical purposes, such as evaluating and monitoring treatment success.
